# Exploring the Interplay of Movement Dispersion, Sleep, and Quality of Life Among Men With and Without HIV

**DOI:** 10.1007/s10461-026-05293-1

**Published:** 2026-08-31

**Authors:** Melissa M. Klamm, Kelley Pettee Gabriel, Jung Hye Sung, Yonghua Yan, Angela Duck, Jennifer A. Schrack, Adam P. Spira, Valentina Stosor, Roger Detels, Sanjay R. Patel, Margaret A. Fischl, Stephen J. Gange, Deborah Konkle-Parker

**Affiliations:** 1 School of Nursing, University of Mississippi Medical Center, 2500 N. State St., Jackson, MS 39216, USA; 2 School of Public Health, University of Alabama at Birmingham, Birmingham, AL, USA; 3 Department of Epidemiology and Biostatistics, Jackson State University, Jackson, MS, USA; 4 Department of Mathematics and Statistical Sciences, Jackson State University, Jackson, MS, USA; 5 Johns Hopkins University, Baltimore, MD, USA; 6 Feinberg School of Medicine, Northwestern University, Chicago, IL, USA; 7 Fielding School of Public Health, University of California, Los Angeles, Los Angeles, CA, USA; 8 School of Medicine, University of Pittsburgh, Pittsburgh, PA, USA; 9 Miller School of Medicine, University of Miami, Miami, FL, USA; 10 Schools of Nursing, Medicine, and Population Health, University of Mississippi Medical Center, Jackson, MS, USA

**Keywords:** Physical activity, Patterns, Movement, Sleep, HIV, MWCCS

## Abstract

Pure and total movement dispersion are measures of the spread of physical activity (PA) across the day, without (pure) and with (total) intensity. Men with HIV (MWH) tend to have lower PA, more sedentary behavior, and poorer sleep quality and quality of life than men without HIV (MWOH). This study explores the relationship of pure and total movement dispersion with sleep quality, total sleep time, and quality-of-life (QoL) among MWH and MWOH. This study was a secondary analysis from the MACS/WIHS Combined Cohort Study (MWCCS). Participants wore an ActiGraph and an Actiwatch accelerometer on their wrist for 7 days to measure PA and sleep and completed the Pittsburgh Sleep Quality Index and the Medical Outcomes Study Short Form. Spearman’s Rank Correlation and multivariable linear regressions were used to analyze relationships among variables. 594 participants were included (M_age_ = 58.1, 55% MWH, 62% White). MWH demonstrated higher pure (Z = − 3.893, *p* < 0.001) and total movement dispersion (Z = − 3.826, *p* < 0.001) than MWOH. MWH reported worse sleep quality (t = − 3.030, *p*=.003) and general QoL (t = 2.942, *p* = 0.003), yet better health compared to last year (t = − 2.557, *p* = 0.011) than MWOH. Pure movement dispersion was significantly associated with less total sleep time (*B* = − 1.227, *p* < 0.001), and total movement dispersion was significantly associated with a higher global general health (*B* = 7.498, *p* = 0.044). Movement across the day, with and without regard to intensity, warrants exploration on whether the way an individual achieves PA is dispersed matters more than the accumulated minutes.

## Introduction

Current physical activity (PA) recommendations for adults are 150 min or more of moderate to vigorous intensity aerobic activity (MVPA) per week and at least two days per week of resistance training, including all major muscle groups [1]. Additionally, maintaining optimal sleep duration and quality promotes overall health. The recommended sleep duration for adults ranges from 7 to 9 h, with 6 h being considered acceptable and less than 6 h being deemed inadequate [2, 3]. Sleep quality primarily refers to an individual’s ability to fall asleep and stay asleep [4]. PA and sleep are potentially modifiable health behaviors that can reduce the incidence of noncommunicable diseases and contribute to the management of existing diseases [5].

People with HIV (PWH) have been shown to perform less PA, have more prolonged sedentary time, and experience more impaired sleep quality than people without HIV (PWOH) [6–9]. Previous studies using accelerometry found that PWH demonstrated lower total activity levels and energy expenditure during the day, along with greater recumbent time [7, 8]. This prevalent behavioral phenotype among PWH may be due to disease complications, including fatigue, infections, poor nutrition, and inflammation [7]. According to two cross-sectional studies of sleep measurement in PWH and people without HIV (PWOH) using wrist-worn accelerometers, PWH did not show a significant difference in total sleep time (TST) compared to PWOH [10], but PWH did exhibit delayed and less robust sleep [11]. Additionally, PWH have been shown to have more impaired sleep quality than PWOH, including difficulty falling and staying asleep, with a prevalence almost six times that of the general U.S. adult population [10]. By measuring aspects of both PA and sleep, researchers can explore the individual and combined effects of these behaviors on various health outcomes from a 24 h movement perspective, providing a more holistic view of the individual’s daily patterns.

**Fig. 1:**
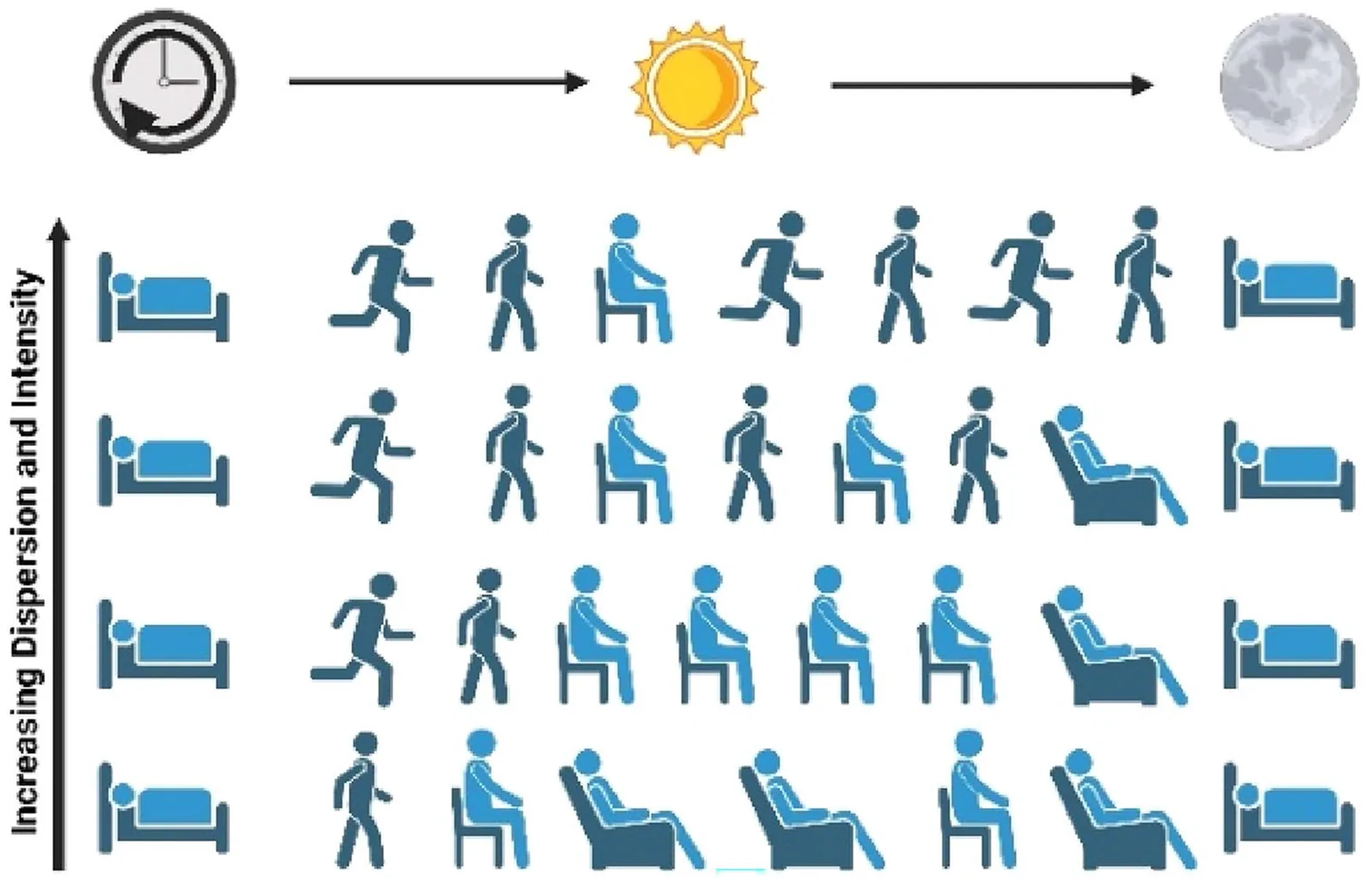
Illustration of pure and total movement dispersion

The daily patterns of PA provide a method for examining the way PA is accumulated, rather than simply reporting total minutes of MVPA [12]. The development of a novel PA variable from raw accelerometer data, called movement dispersion, measures the variability of PA movement across the accelerometer wear time (daytime only). By solely focusing on daytime patterns of movement dispersion, the relationships with nighttime behaviors, specifically sleep, can be kept separate to avoid overlap in analyses. Specifically, total movement dispersion represents both intensity and fragmentation of movement, whereas pure movement dispersion refers explicitly to movement fragmentation, independent of intensity [13]. Figure 1 depicts the range of movement dispersion by illustrating combinations of daily movements with increasing movement and intensity. The bottom line shows a person with minimal movement, mostly sitting or reclining throughout the day, which would represent the lowest end of pure and total movement dispersion. The second line shows a person with more intense movement early in the day, such as bouts of running, followed by minimal movement for the rest of the day, which would relate to a slightly higher total movement dispersion but minimal change in pure movement dispersion. The third line shows movement interspersed with sitting, mostly walking or light-intensity activity, which demonstrates a higher pure movement dispersion by adding movement more regularly despite the intensity of the movement. The top line shows a person with minimal sitting during the day and more intense movements (the running figure) at different times, indicating the highest total movement dispersion. The pure movement would not be altered by the increasing intensity of the activity; only total movement would capture this change. Therefore, the images in the figure that simply show any increase in the frequency of transitions from sitting to moving demonstrate an increase in pure movement dispersion. However, the frequent changes in movement that involve more intense movements, such as running, demonstrate increasing total movement dispersion. Understanding the concept of movement dispersion provides additional nuances about a person’s PA behaviors that may provide novel insights into health outcomes, including nuanced associations of PA with sleep quality and duration and health-related quality of life (HR-QoL) among PWOH and PWH.

HR-QoL, which can include perception of health, physical functioning, fatigue, and pain, can be impacted by sleep duration and quality and the amount of MVPA [14, 15]. With improvement in antiretroviral medications, PWH are living longer, so a new focus has developed on improving HR-QoL beyond simply HIV management, especially as the population ages [16, 17]. Previous studies among PWH have shown lower HR-QoL than among PWOH, which may be explained by disease severity, particularly physical limitations, or social challenges [18, 19]. However, the influence of movement dispersion and sleep on HR-QoL has not been investigated, and this may help identify potential interventions targeting these health behaviors in PWH that improve HR-QoL.

Therefore, the purpose of this cross-sectional study is to (1) describe the patterns of PA, specifically pure and total movement dispersion, among men with (MWH) and without HIV (MWOH), (2) estimate the associations of pure and total movement dispersion with sleep quality, sleep duration, and HR-QoL, while also examining the differences in associations by HIV status, (3) estimate the associations of sleep quality and sleep duration with HR-QoL, and (4) determine if pure or total movement dispersion moderate the relationship between sleep quality or sleep duration and HR-QoL.

## Methods

### Sample

The data were collected as part of the Multicenter AIDS Cohort Study (MACS). The MACS was a longitudinal cohort study that started in 1984 to explore the progression and treatment of HIV and its impact on men’s lives. The study enrolled more than 7300 men who have sex with men with HIV and comparable men without HIV since it began [20, 21]. Study visits occurred every six months at four sites around the United States (Baltimore, Maryland; Chicago, Illinois; Los Angeles, California; and Pittsburgh, Pennsylvania). At each visit, participants completed an in-depth interview, several surveys, underwent a physical examination, and contributed blood and other specimens for laboratory analysis and storage in a national repository that can be accessed with approval to address various HIV-related questions. In 2019, MACS combined with a similar study of women with and without HIV, the Women’s Interagency HIV Study (WIHS), to form the MACS/WIHS Combined Cohort Study (MWCCS) [22].

Occasionally, MACS participants were offered opportunities to participate in ancillary studies related to HIV that required additional data collection outside of the main study visits. From March 2018 to June 2019, MACS participants were given the opportunity to enroll in a sleep study that included both device-based and self-report measures of sleep to detect possible sleep disorders [23]. Individuals who chose to participate in this study completed surveys about sleep quality and an at-home polysomnography (PSG; Nox A1 monitor). In addition, they were instructed to wear a Phillips Actiwatch Spectrum Plus and an ActiGraph GT9X Link accelerometer simultaneously on their non-dominant wrist for seven consecutive days for 24 h each day, to measure sleep duration and PA, respectively. The results from the study’s sleep measures, specifically the PSG, have been reported elsewhere [23, 24]. The Institutional Review Boards at each participating site approved the study activities, and all participants provided informed consent for the stored data to be utilized in future MWCCS-approved studies. Only participants enrolled in the sleep study with valid actigraphy data for both sleep and PA and a MWCCS visit within six months of the accelerometer measurements were included in this study. Individuals with missing demographic data, BMI, inadequate accelerometer wear time, or incomplete surveys from the corresponding MWCCS visit were excluded from analyses.

## Measures

### Physical Activity, Sedentary Behavior, and Movement Dispersion

The Actigraph GT9X Link accelerometer was initialized to sample raw acceleration data at 80 Hz, which was subsequently reintegrated into a 60 s epoch. Data were scored in ActiLife 6 (Ametris, Pensacola, Florida). Briefly, data were screened for non-wear using Choi algorithm [25], and then Montoye et al. [26] thresholds were applied to the minute-level vector magnitude counts to estimate daily time spent sedentary and in light, moderate, and vigorous intensity physical activity. Further, Montoye et al. [26] threshold cut points were used to derive the average duration in minutes of bout estimates for sedentary time, greater than 15 min, and MVPA longer than two minutes after removal of non-wear time. Daily summary estimates for wake time only were averaged across all valid days (≥ 10 h of waking wear on ≥ 3 of 7 days). Pure and total movement dispersion were calculated for daytime hours (0600-2200) from the raw data using a supercomputer. The pure and total movement dispersion algorithms are published elsewhere [13]. Total movement dispersion reflects the intensity of motion by incorporating both the magnitude of the acceleration vector and the temporal distribution of movements. Specifically, it accounts for acceleration magnitudes across different time points, allowing intensified movements to be represented by larger acceleration values—thereby increasing the overall dispersion. In contrast, pure dispersion excludes the magnitude component and focuses solely on the spread of movement, capturing the distribution pattern without regard to intensity. Pure and total movement dispersion are unitless variables, as the units cancel out during calculations, allowing for easier comparisons between data from different groups [13].

### Self-Report Sleep Quality

The Pittsburgh sleep quality index (PSQI) [27], was used to measure sleep quality over the past month. The PSQI consists of 19 self-report questions that produce seven components: sleep quality, sleep latency, sleep duration, habitual sleep efficiency, sleep disturbances, use of sleep medications, and daytime dysfunction. The seven components are scored equally, ranging from 0 to 3, and are summed to obtain a global score, ranging from 0 to 21, where higher scores indicate worse sleep quality. PSQI global scores greater than 5 are considered indicative of poor sleep quality. Cronbach’s α for the PSQI components is 0.83 [27]. The PSQI is widely used in studies to evaluate sleep quality among all ages and different populations, including the elderly and PWH [10, 28].

### Sleep Actigraphy

The participants wore an Actiwatch Spectrum Plus actigraph (Phillips Respironics, Bend, OR) on the non-dominant wrist for seven days and nights, except during prolonged water exposure. This device utilizes a micro-electro-mechanical system (MEMS)-type accelerometer to measure accelerations at a sampling rate of 32 Hz. For this study, the devices were set for 30 s epochs, with activity, light, and marker selections set at default. The participants were instructed to press a button on the device to create event markers for when they went to sleep and woke up each day. The device was returned to the associated study site for data to be downloaded and analyzed through Actiware software (Version 6.0; Phillips Respironics, Bend, OR). Only participants with a minimum of five nights of data were considered valid to ensure accurate sleep estimates [29, 30]. Participants with fewer than five nights were asked to wear the device again within two weeks of being notified that the first attempt failed. The Actiwatch provided a direct measurement of sleep duration, derived from the Actiwatch software using established algorithms to classify rest periods as sleep or awake [31, 32]. The total sleep time (TST) was calculated as the average sleep duration across multiple nights and reported in hours.

### Health-Related Quality of Life

The MACS sleep study used the Medical Outcomes Study Short Form-36 (MOS SF-36) to measure health-related quality of life (HR-QoL). MOS SF-36 contains 36 items that address eight domains of health-related quality of life (physical function, pain, limitations to role function, social function, emotional well-being, and vitality) and a single item for health transition (perception of health compared to 1 year ago) [33, 34]. The subscales can also be combined to reflect overall physical and mental health; however, this study focused on the subscales to capture more specific associations. The items are answered using Likert scales and then recoded as 0–100 according to the MOS scoring guidelines, and then the average is taken from items within each subscale domain to create subscale scores (https://www.rand.org/health-care/surveystools/mos/36-item-short-form/scoring.html) [34]. A higher score represents a more favorable HR-QoL for the associated domain. The MOS SF-36 has been widely used in HIV-associated studies and has demonstrated Cronbach’s α coefficients of over 0.7 for all subscales among this population [33].

### Statistical Analyses

All statistical analyses were completed using IBM SPSS 31 and SAS 9.4. The significance level was set a priori at *p* < 0.05, two-sided. Normality tests were run for the continuous estimates. The demographic data (age and BMI) and outcome measures for sleep duration, sleep quality, and HR-QoL were normally distributed. However, the PA continuous variables were skewed, leading to primarily nonparametric tests for the analyses. Frequency and means were run to analyze the group’s characteristics overall and among the two subgroups, MWH and MWOH. For categorical variables, chi-squared tests were conducted to assess differences between groups. For continuous demographic characteristics (age and BMI) and outcome measures, including sleep and HR-QoL, t-tests were performed to assess the group differences between MWH and MWOH. Mann–Whitney U tests were used to determine the group differences among the PA continuous variables. Spearman’s correlation was used to evaluate the associations between pure and total movement dispersion and measures of sleep and HR-QoL. After log-transforming pure and total movement dispersion, multivariable linear regression models were performed to further assess these relationships, after controlling for HIV serostatus, age, BMI, income level, and education level. Moderation analyses were conducted to examine whether pure or total movement dispersion moderated the relationship between sleep quality or sleep duration and the HR-QoL measures, using interaction terms between sleep quality and movement dispersions, after controlling for HIV serostatus, age, BMI, income level, and education level.

## Results

**Fig. 2:**
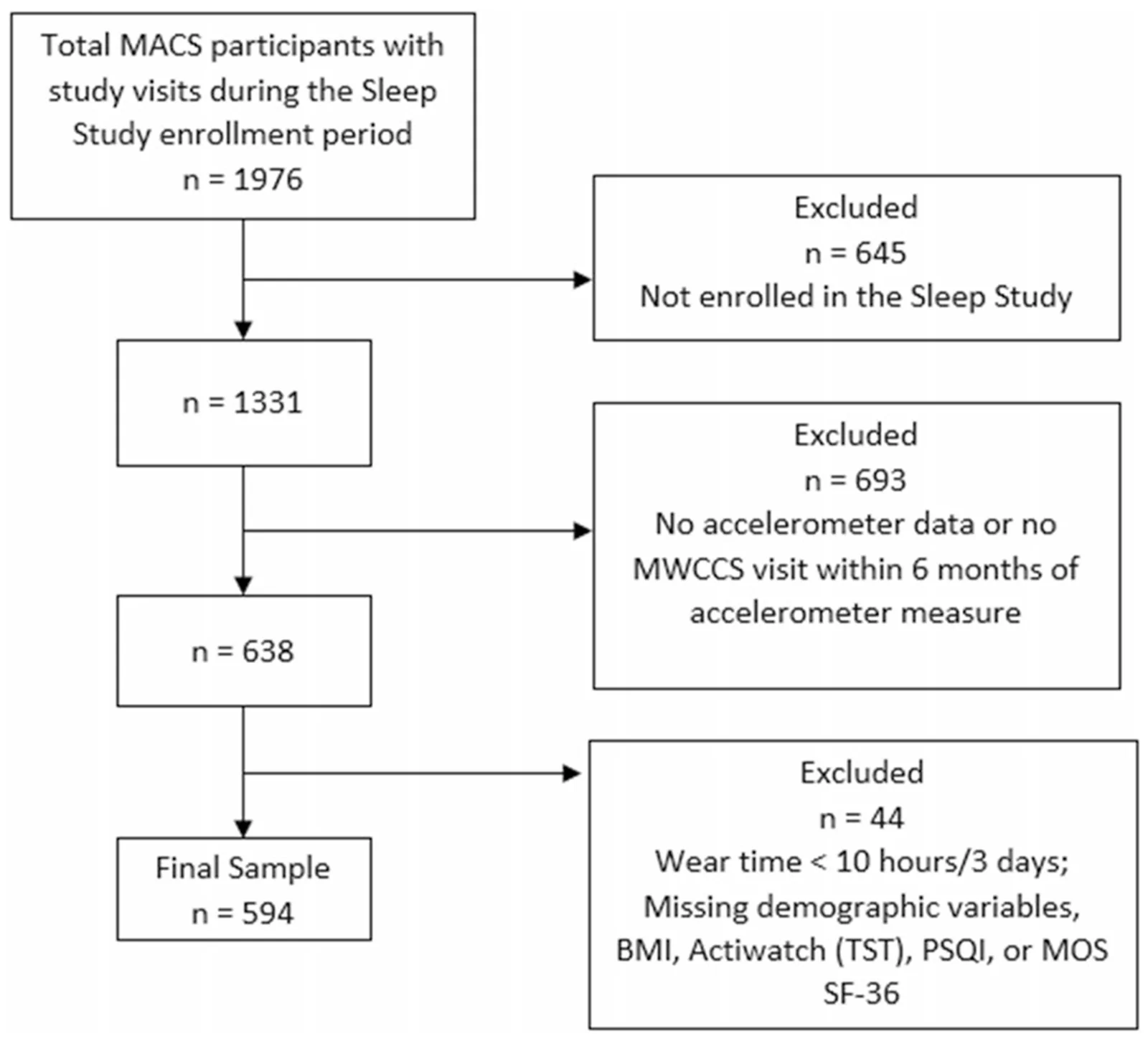
Sample flow diagram. MACS multicenter AIDS cohort study, BMI body mass index, TST total sleep time, PSQI Pittsburg sleep quality index, MOS SF-36 medical outcomes study short form

A total of 1331 men in MACS enrolled in the sleep study. Demographic and other outcome variables were obtained from MWCCS for sleep study participants who had a visit within 6 months of completing the accelerometer measurement (n = 638). After reviewing the data for incompleteness, including accelerometer measures, surveys, and demographics, a final sample of 594 participants was included in this secondary analysis (Fig. 2). Among the 44 participants with collected data from MWCCS who were removed, 20 were PWH and 24 POWH. The removed participants did not differ statistically from the final sample on any demographic variable (Supplemental Table 1). The only variable that differed statistically was average minutes of MVPA, which was lower among those who were removed (removed = 60 ± 45.8; final = 71.47 ± 37.16; Z = 2.69, *p* = 0.007).

The average age of the participants was 58.1 years (SD = 11.4; range = 26–86). Total group demographic characteristics and by HIV serostatus are outlined in Table 1. A statistically significant difference was noted between MWH and MWOH in all demographic variables, except BMI.

Several significant differences were observed in sleep quality, sleep duration, and HR-QoL between MWH and MWOH. MWH demonstrated a nonsignificant lower TST in hours, as measured by the Actiwatch (t = 1.903, *p* = 0.058), than MWOH; however, the total group showed an average TST of approximately 6.2 h, which is below the recommended amount and at the lower limit considered acceptable for sleep duration [2]. PSQI global score for the total group was above five, which is regarded as poor sleep quality [27]. However, MWH had a significantly higher PSQI global score (t = − 3.030, *p* = 0.003) and sleep quality component score (t = − 2.135, *p* = 0.033) than MWOH. MWH also demonstrated significantly lower HR-QoL scores than MWOH scores, specifically related to general health rating, social function, pain, physical limitations on role functioning, and global general health. However, MWH had a significantly higher score on the perception of health compared to one year ago (t = − 2.557, *p* = 0.011).

Table 2 presents the comparison of PA measurement outcomes between MWH and MWOH. Within our sample, MWH demonstrated significantly greater median daily minutes of MVPA (Z = − 2.893, *p* = 0.004) and shorter bouts of sedentary behavior (Z = − 2.697, *p* = 0.007) than MWOH. Additionally, MWH also demonstrated more pure movement dispersion (Z = − 3.893, *p* < 0.001) and total movement dispersion (Z = − 3.826, *p* < 0.001) compared to MWOH. However, the difference between MWH and MWOH remained significant for pure movement dispersion (*B* = − 0.038, *p* = 0.004) and total movement dispersion (*B* = − 0.045, *p* = 0.02) but not MVPA minutes (*B* = − 0.014, *p* = 0.445), MVPA bout length (*B* = 0.009, *p* = 0.221), SB minutes (*B* = − 0.007, *p* = 0.188), or SB bout lengths (*B* = 0.013, *p* = 0.328), after controlling for age using linear regression on log-transformed PA measures. Age demonstrated a significant negative association with pure movement dispersion (*B* = − 0.0017, *p* = 0.004), total movement dispersion (*B* = − 0.0053, *p* < 0.001), average MVPA minutes per day (*B* = − 0.0078, *p* < 0.001), and average MVPA bout length (*B* = − 0.0021, *p* < 0.001), where an increase of one year in age was associated with a decrease in MVPA minutes and bout lengths and pure and total movement dispersion. In addition, age had a significant positive association with the average SB minutes per day (*B* = 0.0013, *p* < 0.001) and average SB bout length (*B* = 0.0043, *p* < 0.001), with an increase of one year in age associated with increases in SB minutes and bout length.

Pure (without intensity included) and total (with intensity) movement dispersion had differential relationships with sleep duration (total sleep time [TST]) and sleep quality (PSQI) among the total sample. Pure movement dispersion demonstrated a significant inverse association with TST (*r*_s_= − 0.167, *p* < 0.001) (Table 3). Total movement dispersion had a similar yet weaker correlation with TST (*r*_s_= − 0.098, *p* = 0.017). However, neither pure nor total movement dispersion was significantly associated with the PSQI global score or single-question on sleep quality rating.

Multivariable linear regressions were run using log-transformed pure movement dispersion and sleep outcomes (PSQI scores and TST), as well as log-transformed total movement dispersion and sleep outcomes. Only the regression model between pure movement dispersion and TST had a statistically significant result when controlled for serostatus, age, education, income, and BMI (Table 4). Pure movement dispersion (dispersion without intensity considered) was significantly associated with lower TST (*B* = − 1.227, *p* < 0.001), where a 1-unit increase in pure movement dispersion was associated with a 22% decrease in TST. Age (*B* = − 0.018, *p* = 0.002), less than a high school education (*B* = − 0.641, *p* = 0.001), and income (*B* = 0.082, *p* = 0.001) were additional covariates with significant associations to lower TST when added to the model for pure movement dispersion. Total movement dispersion (dispersion as well as intensity) demonstrated a nonsignificant association with TST, although in the same inverse direction (*B* = − 0.4305, *p* = 0.095).

When unadjusted, pure and total movement dispersion were significantly associated with a few of the HR-QoL subscales and items (Table 5). Pure and total movement dispersion were significantly associated with the perception of improved health compared to a year ago, with total movement dispersion demonstrating a stronger association. Total movement dispersion also displayed a weak yet statistically significant association with improved physical function and global general health.

These relationships were further examined using multiple linear regression analyses with the log-transformed versions of pure and total movement dispersion and controlling for HIV status, age, education level, income, and BMI. The only relationship that remained significant was total movement dispersion and global general health (Table 6). Total movement dispersion demonstrated a significant association with higher global general health (*B* = 7.498, *p* = 0.044), where a 1-unit increase in total movement dispersion was associated with a sevenfold increase in global general health. Additionally, HIV-negative serostatus, age, income, and BMI were significantly associated with global general health.

Pure movement dispersion demonstrated approaching significance in relation to global health (*B* = 9.861, *p* = 0.064). The relationships with pure movement dispersion and health compared to one year ago were no longer significant in the face of these covariates (*B* = 6.063, *p* = 0.261), yet age (*B* = − 0.352, *p* < 0.001), less than a high school education (*B* = 10.259, *p* < 0.001), and income (*B* = 1.284, *p* < 0.001) were significant. Total movement dispersion displayed a similar relationship to health compared to one year ago when adjusted for covariates (*B* = 3.803, *p* = 0.314), and the same covariates were significantly associated: age (*B* = − 0.339, *p* < 0.001), less than a high school education (*B* = 10.266, *p* < 0.001), and income (*B* = 1.271, *p* < 0.001). For the relationship between total movement dispersion and physical function, it was no longer significant (*B* = 2.974, *p* = 0.459), and, instead, age (*B* = − 0.628, *p* < 0.001), less than a high school education (*B* = − 7.091, *p* = 0.021), income (*B* = 3.594, *p* < 0.001), and BMI (*B* = − 0.661, *p* < 0.001) became the significant covariates associated with health compared to 1 year ago.

Spearman’s Rho correlations of sleep duration and sleep quality with HR-QoL were also analyzed before testing to see if pure or total movement dispersion moderated this relationship. A longer TST was significantly associated with general health, physical function, emotional well-being, social function, physical role limitations, and global general health (all *p* < 0.05). The PSQI global score and single-question sleep quality rating exhibited a statistically significant inverse relationship with all HR-QoL variables (all *p* < 0.001), where a lower PSQI score—indicating better sleep quality—was associated with higher HR-QoL scores. However, none of the potential moderation models between pure or total movement dispersion and sleep (duration or quality) on HR-QoL were significant. This finding indicates that pure and total movement dispersion did not appear to moderate the association between sleep duration or quality and HR-QoL.

## Discussion

Contrary to previous work [6–8], the MWH in this study demonstrated significantly more average minutes of MVPA and shorter sedentary bout lengths than the MWOH. Additionally, MWH had significantly more pure and total movement dispersion than MWOH, which does align with the observed differences in MVPA minutes and SB bouts. The results showed a decline in MVPA and in pure and total movement dispersion, and an increase in SB with age; however, MWH demonstrated significantly greater pure and total movement dispersion despite age. These findings may be due to a heightened awareness among MWH of importance of PA and daily movement in reducing the impact on their physical function and potential comorbidities, such as cardiovascular disease or diabetes. This study did not collect information on current employment status, but such information, if gathered, could provide more insight into differences in pure and total movement dispersion, since men with full-time jobs may have less time to incorporate movement into their day. Other aspects not explored in this study, specific to MWH, include the anti-retroviral treatment (ART) regimen, duration of HIV diagnosis, and viral suppression. These factors would help elucidate the severity and control of the disease among MWH, which, if controlled for in a regression model, could have shown any differences in MVPA by disease severity or treatment regimen [7]. Plus, the MWH in this sample may demonstrate survivor bias by remaining in the cohort study and enrolling in additional substudies, such as the sleep study, since healthier MWH may be more likely to remain in the study and, as a result, align with higher physical functioning and MVPA.

In this study, MWH demonstrated less total sleep time (TST) in hours compared to MWOH, which aligns with previous research findings [9, 10]. The difference between the two groups was near statistical significance; however, MWH had just over six hours of TST, which is the cutoff for adequate sleep according to the National Sleep Foundation recommendations [2]. A shorter sleep duration may be due to HIV disease symptoms, such as pain, or simply a result of difficulty falling asleep, since MWH reported a significantly worse sleep quality than MWOH. People with HIV (PWH) have been found to have a more impaired sleep quality compared to the general population, with almost 60% of PWH having reported some form of sleep disturbance, including insomnia or sleep-disordered breathing [10, 23, 24].

Similarly, MWH overall reported significantly worse HR-QoL in most areas compared to MWOH, except for perceptions of health compared to one year ago, which MWH reported as significantly higher than MWOH. However, no significant difference was noted related to physical function or fatigue, which previous studies cited as possible reasons for the disparities they found in HR-QoL [18, 19]. Among participants in this study, the MWH reported significantly worse general and overall health, pain, emotional well-being, social functioning, and physical role limitations.

After adjusting for potential confounders, pure movement dispersion was no longer significantly associated with any of the HR-QoL outcomes, and total movement dispersion was only significantly associated with global general health. This finding demonstrates that more intense movement spread throughout the day is associated with improved overall health perceptions, even in the face of advancing age and higher BMI. However, HIV-negative serostatus, younger age, higher education, lower BMI, and higher income also demonstrated significant associations with higher global general health in this model, with total movement dispersion. Therefore, it is important to consider these other aspects when developing interventions to increase more intense daily movement patterns for improving HR-QoL.

Pure movement dispersion was significantly associated with lower TST, but total movement dispersion was not. A greater amount of movement spread across the day may have affected the amount of time allocated for sleep each day. In the same way, the reduced time spent sleeping at night might have facilitated a broader time for PA, which demonstrates the bidirectional relationship between PA and sleep [35]. This point is supported by the displacement hypothesis, which states that more time spent on one behavior reduces the time available for another behavior within the same 24 h [36]. However, these results cannot be interpreted as causal due to their cross-sectional nature, so although the behaviors demonstrate a negative association, other aspects of daily life could be altering the relationship, such as the difference between leisure and occupational PA or variations in light exposure, which were not captured in this study [37–39]. Previous studies have focused primarily on exercise as reported by MPVA minutes; however, pure movement dispersion does not capture intensity, only general movement. No studies examining the spread of PA across the day and TST were identified, both cross-sectional and longitudinal. However, Kuldas et al. [36] explored within-person variations in PA, SB, and sleep in a longitudinal study of children as they transitioned from age 6 to 18. Their results showed that total PA, MVPA, and SB were inversely associated with TST, similar to our study, and within-person changes over time in these three areas did not predict changes in TST. Conversely, a study that followed sleep and activity patterns of women over 21 days found that on days when they accumulated more PA than on average, they had longer TST [40]. The results from this study do align with previous studies showing that lower TST was associated with higher daily MVPA minutes, and that more intense vigorous PA was associated with shorter TST [35, 41]. Hofman et al. [42] found that replacing 30 min of sleep with MVPA was associated with shorter sleep duration, and more SB was associated with poorer sleep quality. However, these studies did not examine the associations of PA, SB, and TST, specifically among PWH. Possible explanations for the effect of PA on sleep include improved thermoregulation, increased parasympathetic activity, and psychosocial factors, but no definitive cause has been concluded [36, 38, 39]. Additionally, pure movement dispersion does not account for intensity and therefore may play a different role than strictly MVPA, or it may not affect sleep duration in the end. Future work should explore the impact of pure and total movement dispersion, including within-person variation, on TST across multiple time points.

Although neither pure nor total movement dispersion demonstrated statistically significant moderation of the relationship between sleep quality or sleep duration and HR-QoL, these findings remain informative. The lack of moderation may have resulted from the strength of the association between sleep (quality and duration) and HR-QoL, since these associations were much stronger than those between pure and total movement dispersion and HR-QoL. Additionally, the sample size may not have been large enough to detect the moderation, since such effects are often smaller and require larger sample sizes to achieve power than direct association analyses. The sample of this study only included men with HIV or at risk of HIV, which may have reduced the heterogeneity and decreased the ability of the study to detect differential effects. The cross-sectional design can also limit the ability to detect influences. Liang et al. [43] examined objective measures of PA and sleep among participants in the UK Biobank cohort study, with a sample of almost 90,000 men and women, and found that total PA and MVPA attenuated the effects of inadequate or excessive TST on cardiovascular disease mortality risk. Although pure and total movement dispersion measure activity in different ways, future studies should explore the ability of these variables to mitigate the effects of poor sleep quality on similar health outcomes, specifically in a longitudinal study with a large sample of men and women.

The limitations of this study include its cross-sectional nature, which limits the interpretability of the relationship between pure and total movement dispersion and sleep and HR-QoL. The participants also wore the accelerometers on their wrists, and previous studies have demonstrated that wrist placement may overestimate the minutes in MVPA [44]. However, the consistency of placement for measurement across all participants would reduce the impact of an overestimation on differences noted between MWH and MWOH. Additionally, the sleep quality and HR-QoL outcomes were assessed using self-report tools, which may be influenced by social desirability bias or recall limitations. The results should be interpreted with caution for generalizability to only similar populations of MWH or men at risk for HIV, not the general population.

Although this study found only weak associations with HR-QoL, the associations between pure and total movement dispersion and health outcomes may not have been detected using the cross-sectional approach. With these initial findings linking pure and total movement dispersion to positive HR-QoL outcomes, future studies should explore these relationships, along with sleep outcomes, further in a longitudinal study to determine the potential influence of pure and total movement dispersion on health over time. Such findings would help promote the use of these measures as outcome variables in future studies and translate into interventions to increase movement throughout the day.

## Conclusion

Movement across an individual’s day, with and without regard to intensity, is an important aspect of PA that needs to be explored to determine whether the way an individual achieves PA matters more than the accumulated minutes per day or week. Pure and total movement dispersion did not have a positive relationship with perceived sleep quality or duration. In the partially adjusted models, total movement dispersion, which accounts for movement intensity, was associated with physical function. However, the association became statistically null in the fully adjusted models. In contrast, total movement dispersion remained significantly associated with global general health in the fully adjusted model. Future work should include a larger, more diverse sample and focus on measuring outcomes longitudinally to detect potentially dynamic influences of pure and total movement dispersion on health.

Pure and total movement dispersion are not intended to replace established measures, such as minutes in MVPA, but rather to complement them, providing a richer description of an individual’s PA patterns and, therefore, offering more insights into the relationship of PA accumulation and health. These measures of movement dispersion may be more predictive than traditional measures in explaining how PA patterns relate to health over time, especially among MWH who are at greater risk of non-communicable diseases, such as cardiovascular disease and diabetes, than the general public. Therefore, future work should explore the impact of pure and total movement dispersion on health outcomes among people with and without HIV.

## Supplementary Material

Supplemental Table 1

The online version contains supplementary material available at https://doi.org/10.1007/s10461-026-05293-1.

## Figures and Tables

**Table 1:** Comparison of demographics and outcomes among total group and by serostatus

	Total sample (n = 594)	MWH (n = 328)	MWOH (n = 266)	Test statistic (*χ*^2^/t)	*p*
	M ± SD/n (%)	M ± SD/n (%)	M ± SD/n (%)		
Age (years)	58.1 ± 11.3	55.9 ± 10.6	60.9 ± 11.7	t = 5.403	< 0.001**
Race				*χ*^2^ = 27.804	< 0.001**
American Indian/Alaskan Native	20 (3.4%)	16 (4.9%)	4 (1.5%)		
Asian	2 (0.3%)	2 (0.6%)	–		
Native Hawaiian/Pacific Islander	3 (0.5%)	2 (0.6%)	1 (0.4%)		
Black	161 (27.1%)	108 (32.9%)	53 (19.9%)		
White	369 (62.1%)	176 (53.7%)	193 72.6%)		
Other	6 (1%)	3 (0.9%)	2 (0.8%)		
Multiracial	33 (5.6%)	22 (6.7%)	11 (4.1%)		
Ethnicity					
Hispanic	88 (14.8%)	63 (19.2%)	25 (9.4%)	*χ*^2^ = 11.198	< 0.001**
Education				*χ^2^* = 27.511	< 0.001**
≤ 12th grade	129 (21.7%)	91 (27.7%)	38 (14.3%)		
At least 1 year college	156 (26.3%)	85 (25.9%)	71 (26.7%)		
College degree	140 (23.6%)	79 (24.1%)	61 (22.9%)		
Graduate degree	169 (28.5%)	73 (22.3%)	96 (36.1%)		
BMI	27.3 ± 5.2	26.9 ± 4.9	27.8 ± 5.5	t = 1.901	0.058
Income				*χ^2^* = 27.778	< 0.001**
< $30,000	223 (37.5%)	151 (46%)	72 (27.1%)		
$30,000–59,999	162 (27.3%)	88 (26.8%)	74 (27.8%)		
$60,000–99,999	93 (15.7%)	41 (12.5%)	52 (19.5%)		
≥ $100,000	92 (15.5%)	38 (11.6%)	54 (20.3%)		
Did not answer	24 (4%)	10 (3%)	14 (5.3%)		
TST hours	6.24 ± 1.45	6.14 ± 1.45	6.37 ± 1.43	t = 1.903	0.058
PSQI					
Global score	6.81 ± 3.69	7.23 ± 3.8	6.3 ± 3.48	t = − 3.030	0.003*
Sleep quality component score	1.15 ± 0.74	1.1 ± 0.72	1.08 ± 0.74	t = − 2.135	0.033*
MOS SF-36					
General health rating	62.9 ± 25.1	60.2 ± 25.8	66.3 ± 23.7	t = 2.942	0.003*
Compared to 1 year ago	53.2 ± 21.5	55.3 ± 22.7	50.8 ± 19.7	t = − 2.557	0.011*
Physical function	82.7 ± 24.6	81.42 ± 24.9	84.2 ± 24.3	t = 1.385	0.167
Energy/fatigue	60.58 ± 22.96	59.07 ± 24.87	62.44 ± 20.25	t = 1.784	0.075
Emotional well-being	75.1 ± 19.9	73.7 ± 21.1	76.9 ± 18.4	t = 2.000	0.046*
Social function	83.1 ± 24	79.9 ± 25.8	87 ± 21	t = 3.267	< 0.001**
Pain	77.3 ± 23.3	75.5 ± 24.1	79.6 ± 22	t = 2.154	0.032*
Role limitations-physical	79.1 ± 36.9	75.8 ± 38.4	83.2 ± 34.6	t = 2.446	0.015*
Role limitations-emotional	80.4 ± 35.8	79 ± 36.4	82.2 ± 35	t = 1.095	0.274
Global general health	65.9 ± 21.4	62.6 ± 23.3	69.9 ± 18.3	t = 4.132	< 0.001**

*IQR* interquartile range, *MWH* men with HIV, *MWOH* men without HIV, *M* mean, *SD* standard deviation, *MWH* men with HIV, *MWOH* men without HIV, *TST* total sleep time, *PSQI* Pittsburgh sleep quality index, *MOS SF-36* medical outcomes short form

* *p* < 0.05,

** *p* < 0.001

**Table 2:** Comparison of physical activity measures by serostatus

	Total sample (n = 594)	MWH (n = 328)	MWOH (n = 266)	Z	*p*
	Median (25–75%)/M ± SD	Median (25–75%)/M ± SD	Median (25–75%)/M ± SD		
Avg. min MVPA	65 (44.95–87.78)/71.47 ± 37.16	69.9 (46.18–92.08)/74.9 ± 37.69	59.2 (45.53–82.33)/67.23 ± 36.11	− 2.893	0.004*
Avg. min SB	766.02 (694.34–839.25)/768.02 ± 106.77	769.31 (687.15–840.44)/765.45 ± 109.04	764.42 (698.06–830.34)/764.49 ± 104.11	− 0.356	0.722
Avg. MVPA bout length in min	3.5 (3.1–4)/3.64 ± 0.93	3.6 (3.1+ − 4)/3.62 ± 0.76	3.5 (3.1–4)/3.67 ± 1.11	− 0.832	0.405
Avg. SB bout length in min	89.75 (72.4–115.73)/102.03 ± 51.63	86.4 (70–113.03)/97.65 ± 42.11	95.05 (76.6–118.25)/107.44 ± 61.01	− 2.697	0.007*
Pure movement dispersion	0.035 (0.026-0.045)/0.037 ± 0.013	0.037 (0.028-0.047)/0.039 ± 0.014	0.032 (0.025-0.042)/0.034 ± 0.012	− 3.893	< 0.001**
Total movement dispersion	79.52 (54.99–115.81)/92.54 ± 52.92	86.47 (59.68–124.87)/98.95 ± 54.34	71.24 (50.69–105.64)/84.64 ± 50.11	− 3.826	< 0.001**

*IQR* interquartile range, *M* mean, *SD* standard deviation, *Avg.* average, *min* minute, *MVPA* moderate to vigorous physical activity, *SB* sedentary behavior

**Table 3:** Spearman’s Rho correlation results of pure and total movement dispersion with sleep quality (PSQI) and TST (actigraphy)

Sleep measures	Pure movement dispersion	Total movement dispersion
	*r_s_* (*p*)	*r_s_* (*p*)
Actiwatch		
TST (hours)	− 0.167 (0.001*)	− 0.098(0.017*)
PSQI		
Global score	− 0.006 (0.886)	− 0.042 (0.312)
Sleep quality component score	− 0.02 (0.624)	− 0.063 (0.128)

*TST* total sleep time, *PSQI* Pittsburgh sleep quality index

* *p* < 0.05,

** *p* < 0.001

**Table 4:** Linear regression results of daytime pure movement dispersion as a predictor of total sleep time (TST)

Predictor	Unstandardized *B*	Standard error	Unstandardized 95% confidence interval		Wald χ^2^	*p*
			LL	UL		
Log pure movement dispersion	− 1.227	0.365	− 1.943	− 0.512	11.31	0.001*
HIV-negative serostatus	0.054	0.123	− 0.186	0.295	0.19	0.659
Age	− 0.018	0.006	− 0.029	− 0.007	9.78	0.002*
Education						
≤ High school	− 0.641	0.196	− 1.025	− 0.257	10.71	0.001*
≥ 1 year college	− 0.362	0.168	− 0.692	− 0.032	4.62	0.032*
College degree	− 0.357	0.165	− 0.681	− 0.033	4.67	0.031*
Income	0.082	0.026	0.032	0.132	10.18	0.001*
BMI	0.006	0.011	− 0.016	0.028	0.26	0.608

*BMI* body mass index

* *p* < 0.05,

** *p* < 0.001

**Table 5:** Spearman’s rho correlation results of pure and total movement dispersion with HR-QoL

MOS SF-36	Pure movement dispersion	Total movement dispersion
	*r_s_* (*p*)	*r_s_* (*p*)
Single items		
General health rating	0.013 (0.761)	0.044 (0.285)
Compared to 1 year ago	0.105 (0.01*)	0.126 (0.002*)
Subscale scores		
Physical function	0.006 (0.887)	0.082 (0.045*)
Energy/fatigue	0.023 (0.580)	0.045 (0.269)
Emotional well-being	− 0.019 (0.635)	− 0.037 (0.367)
Social function	0.023 (0.584)	0.009 (0.829)
Pain	0.02 (0.619)	0.054 (0.187)
Role limitations-physical	0.035 (0.401)	0.072 (0.08)
Role limitations-emotional	− 0.001 (0.988)	0.004 (0.914)
Global general health	0.033 (0.419)	0.083 (0.044*)

* *p* < 0.05,

** *p* < 0.001

**Table 6:** Linear regression results of daytime total movement dispersion as a predictor of global general health

Predictor	Unstandardized *B*	Standard error	Unstandardized 95% confidence interval		Wald χ^2^	*p*
			LL	UL		
Log total movement dispersion	7.498	3.721	0.205	14.79	4.06	0.044*
HIV-negative serostatus	5.929	1.781	2.439	9.42	11.09	< 0.001**
Age	− 0.272	0.085	− 0.438	− 0.107	10.39	0.001*
Education						
≤ High school	1.929	2.853	− 3.664	7.521	0.46	0.499
≥ 1 year college	4.36	2.45	− 0.442	9.163	3.17	0.075
College degree	− 1.333	2.404	− 6.045	3.379	0.31	0.579
Income	2.476	0.374	1.743	3.209	43.81	< 0.001**
BMI	− 0.379	0.165	− 0.703	− 0.055	5.26	0.022*

*BMI* body mass index

* *p* < 0.05,

** *p* < 0.001

## Data Availability

Access to individual-level data from the MACS/WIHS Combined Cohort Study Data (MWCCS) may be obtained upon review and approval of an MWCCS concept sheet. Links and instructions for online concept sheet submission are on the study website (http://mwccs.org/).
