# Supplementary material for: Exploring the Interplay of Movement Dispersion, Sleep, and Quality of Life Among Men With and Without HIV
Source: AIDS Behav. Author manuscript; Available in PMC 2026 Sep 21. (PMC13591559; doi:10.1007/s10461-026-05293-1)
Supplement: Supplementary file 1 [file NIHMS2207947-supplement-Supplemental_Table_1.docx]

Supplemental Table 1

*Comparison of Removed Participants with the Final Sample*

|  | Removed  (n = 44) | Final Sample  (n = 594) | Test Statistic  (ꭓ^2^ / Z / t) | *p* |
| --- | --- | --- | --- | --- |
|  | M±SD/  n (%) | M±SD/  n (%) |  |  |
| Age (years) | 57.7 ±12.6 | 58.1 ±11.3 | t = -.257 |  |
| Race |  |  | ꭓ^2^ = 5.374 | .614 |
| American Indian/Alaskan Native | 4 (9/1%) | 20 (3.4%) |  |  |
| Asian | 0 (0%) | 2 (0.3%) |  |  |
| Native Hawaiian/ Pacific Islander | 0 (0%) | 3 (0.5%) |  |  |
| Black | 10 (22.7%) | 161 (27.1%) |  |  |
| White | 26 (59.1%) | 369 (62.1%) |  |  |
| Other | 1 (2.3%) | 6 (1%) |  |  |
| Multiracial | 3 (6.8%) | 33 (5.6%) |  |  |
| Ethnicity |  |  | ꭓ^2^ = .045 | .831 |
| Hispanic | 6 (13.6%) | 88 (14.8%) |  |  |
| Education |  |  | ꭓ^2^ = 2.612 | .856 |
| ≤12^th^ grade | 10 (22.7%) | 129 (21.7%) |  |  |
| At least 1 yr college | 15 (34.1%) | 156 (26.3%) |  |  |
| College degree | 9 (20.5%) | 140 (23.6%) |  |  |
| Graduate degree | 10 (22.7%) | 169 (28.5%) |  |  |
| Income |  |  | ꭓ^2^ = 3.153 | .533 |
| <$30,000 | 22 (50%) | 223 (37.5%) |  |  |
| $30,000-59,999 | 9 (20.5%) | 162 (27.3%) |  |  |
| $60,000-99,999 | 7 (15.9%) | 93 (15.7%) |  |  |
| ≥$100,000 | 5 (6.8%) | 92 (15.5%) |  |  |
| Did not answer | 1 (2.3%) | 24 (4%) |  |  |
| TST (hours) | 6.77 ±1.6 | 6.24 ±1.45 | t = 1.953 | .373 |
| PSQI |  |  |  |  |
| *Global Score* | 7.36 ±3.71 | 6.81 ±3.69 | t = .936 | .493 |
| MOS SF-36 |  |  |  |  |
| *Global General Health* | 70.91 ±18.1 | 65.9 ±21.4 | t = 1.52 | .074 |
| Physical Activity |  |  | Z = 2.69 | .007* |
| MVPA (minutes) | 60 ±45.8 | 71.47 ±37.16 |  |  |

Note: *p* < .05*; MVPA among removed participants: MWH = 73.08 ±55.15; MWOH = 49.12 ±33.59; BMI, pure movement dispersion, and total movement dispersion were not calculated on the removed participants.
